# Cell–Material Interplay in Focal Adhesion Points

**DOI:** 10.1021/acsami.3c19035

**Published:** 2024-02-14

**Authors:** Krzysztof Berniak, Daniel P. Ura, Adam Piórkowski, Urszula Stachewicz

**Affiliations:** † Faculty of Metals Engineering and Industrial Computer Science, AGH University of Krakow, al. A. Mickiewicza 30, Krakow 30-059, Poland; ‡ Department of Biocybernetics and Biomedical Engineering, AGH University of Krakow, al. A. Mickiewicza 30, Krakow 30-059, Poland

**Keywords:** focal adhesion, fibers, scaffold, paxillin, vinculin, AiryScan, cluster analysis

## Abstract

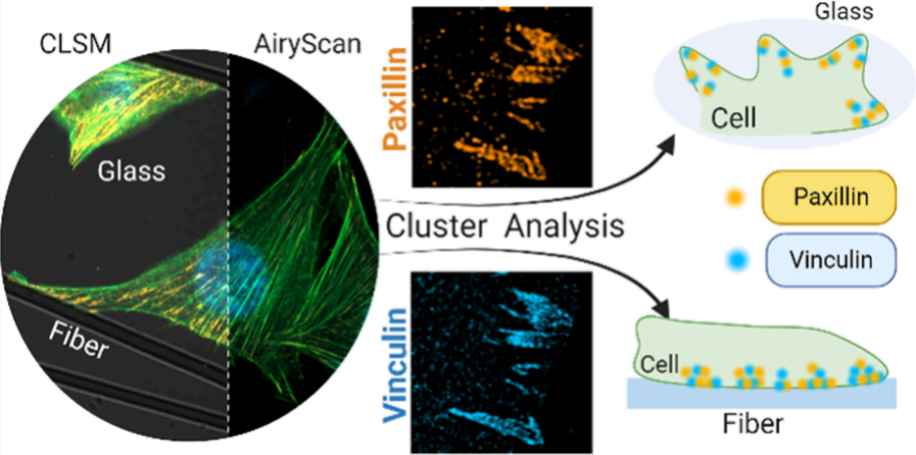

The complex interplay between cells and materials is a key focus of this research, aiming to develop optimal scaffolds for regenerative medicine. The need for tissue regeneration underscores understanding cellular behavior on scaffolds, especially cell adhesion to polymer fibers forming focal adhesions. Key proteins, paxillin and vinculin, regulate cell signaling, migration, and mechanotransduction in response to the extracellular environment. This study utilizes advanced microscopy, specifically the AiryScan technique, along with advanced image analysis employing the Density-Based Spatial Clustering of Applications with Noise (DBSCAN) cluster algorithm, to investigate protein distribution during osteoblast cell adhesion to polymer fibers and glass substrates. During cell attachment to both glass and polymer fibers, a noticeable shift in the local maxima of paxillin and vinculin signals is observed at the adhesion sites. The focal adhesion sites on polymer fibers are smaller and elliptical but exhibit higher protein density than on the typical glass surface. The characteristics of focal adhesions, influenced by paxillin and vinculin, such as size and density, can potentially reflect the strength and stability of cell adhesion. Efficient adhesion correlates with well-organized, larger focal adhesions characterized by increased accumulation of paxillin and vinculin. These findings offer promising implications for enhancing scaffold design, evaluating adhesion to various substrates, and refining cellular interactions in biomedical applications.

## Introduction

Understanding the complex interplay between cells and materials is essential in developing optimal scaffolds to facilitate the regenerative process in the field of regenerative medicine. Currently, this area of study is at the forefront of scientific investigation. Its primary goal is to find solutions for tissue regeneration and cell transplantation scenarios, which are urgent needs.^1^ By gaining insights into the dynamic interactions between cells and scaffolding materials, researchers can strategically design and refine scaffolds to effectively support tissue regeneration, thereby contributing to the advancements in regenerative medicine that are critical for addressing the complex challenges posed by various healthcare conditions.^2,3^

In the realm of investigating cell adhesion within the context of tissue engineering, the selection of an appropriate cell culture substrate plays a pivotal role. Traditional glass substrates, prevalent in laboratory settings, are renowned for their transparency and chemical stability. Nevertheless, inherent drawbacks such as the absence of a three-dimensional (3D) structure and rigidity raise concerns, particularly in endeavors aiming to replicate conditions akin to native tissues. Glass, characterized by its ubiquity and historical prevalence in cell research, boasts advantages in terms of widespread usage and chemical stability. Its optical transparency facilitates high-quality microscopic observations. However, the lack of a 3D structure poses limitations, impeding the faithful recreation of tissue-mimicking conditions. In contrast, polymer fiber scaffolds emerge as a promising alternative, offering the ability to mimic the 3D architecture of native tissues. These scaffolds boast tunable mechanical properties, enhancing their ability to replicate in vivo conditions more accurately.^4^ Their versatility in design and manipulation allows for the tailoring of substrate characteristics to meet specific experimental requirements. While glass maintains its status as a conventional substrate with advantages in transparency and chemical stability, polymer fiber scaffolds provide a biomimetic environment that addresses limitations associated with two-dimensional (2D) substrates. Hence, substrates with a 3D structure, such as fibrous scaffolds, pose greater challenges. Cell adhesion to scaffolds is a critical process in tissue engineering and regenerative medicine. It involves the attachment of cells to surfaces that are often polymer fibers, which can provide structural support and cues for cell proliferation and differentiation.^5−8^ One key aspect of cell adhesion is the formation of focal adhesion (FA) sites, which are specialized structures that link the cell cytoskeleton to the extracellular matrix (ECM). These sites play crucial roles in cell signaling, migration, and mechanotransduction and are essential for the success of tissue engineering applications using electrospun fibers.^9^ These FAs are formed by a complex network of proteins, including integrins, talin, paxillin, and vinculin. Among these proteins, paxillin and vinculin have emerged as key regulators of FA construction and function.^10,11^ Paxillin is an adaptor protein that plays a vital role in the formation and turnover of FAs. It serves as a scaffold for multiple signaling proteins, including integrins, focal adhesion kinase (FAK), and vinculin, all critical components of FAs.^12^ Paxillin helps to organize FAs’ structural and signaling components, regulating their formation, size, and turnover. It also participates in downstream signaling pathways, such as those involving Rho GTPases and MAP kinases, that are important for cell migration and proliferation.^13^ In addition, paxillin can be phosphorylated by FAK and other kinases, leading to changes in its conformation and interactions with other proteins in the FA complex.^14−16^ This phosphorylation can regulate FA dynamics and downstream signaling, further contributing to the overall function of FAs in cell behavior. Another key component of FAs is vinculin, which plays a critical role in regulating their mechanical properties and signaling functions. Vinculin is a cytoskeletal protein that interacts with both actin filaments and integrin receptors, allowing it to link the ECM to the cell cytoskeleton.^17,18^ One of the key functions of vinculin in FAs is to regulate their tension and mechanical properties. Vinculin participates in the stimulation of actin polymerization in response to mechanical stimuli.^19^ It helps to mediate the transmission of mechanical forces between the cell and the ECM, allowing cells to respond to changes in substrate stiffness and other mechanical cues.^20,21^ Vinculin also maintains the structural integrity of FAs, preventing them from collapsing under mechanical stress. In the case of its mechanical functions, vinculin regulates downstream signaling pathways.^22^ Importantly, it interacts with various signaling proteins, including talin, paxillin, and FAK, and can modulate their activities in response to changes in the extracellular environment.^9^ Therefore, in our studies, we focus on these two proteins, paxillin and vinculin, and correlations between them.

Over the past two decades, microscopy techniques have brought about a revolutionary change in the investigation of adhesion sites, crucial components in cell–cell and cell–matrix interactions.^23^ These techniques provide the ability to visualize adhesion sites at a nanoscale level, allowing for a deeper understanding of their structure and function.^24−28^ Super-resolution microscopy, such as structured illumination microscopy (SIM) and stimulated emission depletion (STED) microscopy, represents a sophisticated technique employed for investigating adhesion sites, facilitating the visualization of structures beyond the diffraction limit of light.^29^ These advanced methods have been extensively utilized to explore the organization of FA and the dynamic behaviors of their components. SIM has been used to visualize the organization of FAs, which are large adhesion sites that form at the ends of actin stress fibers.^30^ The use of super-resolution microscopy in the study of adhesion sites showed that dozens of proteins are recruited to adhesion sites, forming nanodomains arranged in three layers. The signaling layer contains integrins that interact with the extracellular environment and with FAK and paxillin proteins. The layer responsible for signal transduction contains talin and vinculin, while the upper layer contains zyxin, vasodilator-stimulated phosphoprotein, and α-actinin.^25^ The layered structure of the adhesion sites has been confirmed for large cornerstone FAs formed in colonies of human pluripotent stem cells.^24^ The super-resolution technique has been used to investigate the movement of microtubule tips near FAs and stress fibers.^31^ Zamir and Geiger postulated that dozens of types of proteins are recruited to adhesion sites.^32^ Moreover, the AiryScan confocal super-resolution method (AiryScan) is an advanced microscopy technique developed by Zeiss (Germany). Similar to conventional confocal microscopes, AiryScan utilizes a laser for single-point excitation. Nevertheless, unlike confocal microscopes, which employ a single photomultiplier detector and a pinhole to eliminate out-of-focus light, the AiryScan is equipped with 32 detectors arranged in a hexagonal array. Each detector functions as an individual pinhole, and the entire array of detectors is employed to calculate the point of origin for all emitted light.^33^ The resolution of the AiryScan image is typically two to three times higher than that of a conventional confocal laser scanning microscope (CLSM).^34^ Therefore, we want to use this microscopy technique to investigate the distribution of paxillin and vinculin in areas where cells bind to the outer matrix. Although AiryScan is a commercially available confocal super-resolution method, it has not been explored on other systems than glass, which, in our case, we are adding to the system polymer fibers used in designing scaffolds for cells. To proceed with the advanced image analysis, an extra effort in developing new protocols and analytical software tools was included. In microscopic images, paxillin and vinculin that have been immunofluorescently labeled form characteristic clusters and aggregates at the adhesion sites. Each focus may consist of several hundred to even a thousand protein molecules of a selected protein. It is interesting to note that despite the fact that these proteins are part of a multiprotein complex (as referenced), their distribution pattern on microscopic images differs at the adhesion sites. Osteoblast cells are used in cell studies focusing on the adhesion process to polymer fibers due to their relevance to bone tissue, natural adhesive properties, and ability to proliferate and differentiate into osteocytes. Their study helps evaluate biomaterial–cell interactions and optimize tissue engineering scaffolds for bone regeneration.

In this study, we focus on quantitatively characterizing the structure of osteoblast cell FA sites and their distribution during connection to the polymer fibers. We examine the molecular distribution of paxillin and vinculin—components of FAs in the binding sites of cells to fibers based on super-resolution microscopic images. We will also explore their roles in cell mechanotransduction and how they interact with the ECM and cytoskeleton. This requires advanced and innovative cluster analysis of proteins within FA sites of cells, extending beyond the 2D environment to encompass a 3D context as well.

**Figure 1:**
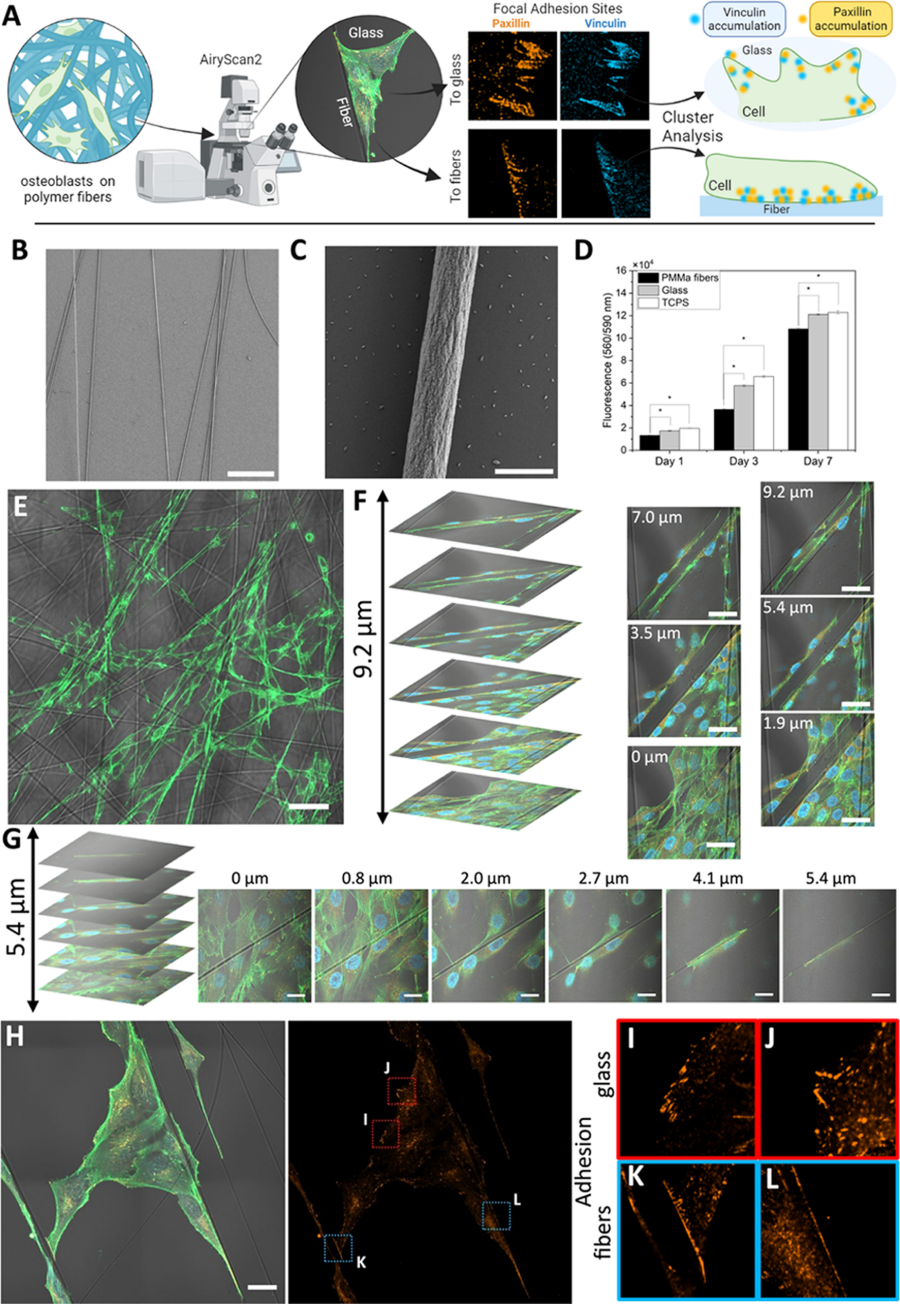
Adhesion of osteoblasts to fibers. (A) Conceptual representation of the research. (B) SEM micrograph of fibers on the glass. Scale bar: 100 μm. (C) SEM image of surface single fiber. Scale bar: 2 μm. (D) Proliferation assays of osteoblast MG-63 cells on fibers, glass, and TCPS as the controls. (E) CLSM image of cells spreading on fibers after 3 days. Scale bar: 50 μm. (F) Z-stacks of CLSM images of osteoblasts surrounding the fiber. Scale bars: 30 μm. (G) Z-stacks of CLSM images of osteoblasts pulled up on the fiber. Scale bars: 20 μm. (H) Cells spreading between two fibers. Additionally, paxillin distribution in a separate channel. Distribution of paxillin at FA sites to glass (zoom in I and J) and fiber (zoom in K and L). Scale bar: 30 μm. On all CLSM images, paxillin was stained with Alexa Fluor Plus 555 (orange), actin fibers were stained with Alexa Fluor 488 Phalloidin (green), and nuclei were counterstained with DAPI. Statistical significance was calculated with ANOVA, followed by Tukey’s post hoc test, *p < 0.05; error bars are based on standard errors.

Nowadays, cluster analysis methods are used to identify spatial patterns and colocalize proteins involved in biological processes within cells or tissues. By applying cluster analysis, researchers can quantitatively assess the spatial relationships between the proteins, determine if they tend to cluster together or segregate, and gain insights into their potential functional interactions or involvement in specific cellular events.^35^ This approach helps to reveal patterns that might not be apparent through visual inspection alone and provides valuable information about the organization and dynamics of the proteins within the biological context under investigation.^36^ Here, we developed novel protocols to perform highly advanced cluster analysis of paxillin and vinculin in FA sites that were explored in connection to the extracellular scaffold and glass. The conceptual representation of the research is shown in Figure 1A. To underline the novelty of this study, it is the first time that the analyses are performed directly on polymer fibers and compared to those commonly used in this type of study glass. Furthermore, we discuss the potential application of a quantitative description of the distribution of adhesion markers in assessing the impact of material on cells. We examine the effects of polymer fibers, FA formation patterns, and the implications for applying scaffolds in tissue engineering and regenerative medicine. Finally, we highlight the challenges and opportunities in the field of cell adhesion to scaffolds’ fibers and FA construction and the potential for these technologies to revolutionize regenerative medicine.

## Results

To verify the effect of polymer fiber building scaffolds, electrospun poly(methyl methacrylate) (PMMA) fibers were employed as the model for quantitative assessment of osteoblast cell adhesion (Figure 1B). The PMMA polymer is extensively utilized in bioengineering as an integral component of bone implants, serving as an adhesive platform for diverse cell types, including osteoblasts. The fibers utilized in the study were fabricated following a previously described methodology.^37^ These fibers exhibited an average diameter of 3.13 ± 0.22 μm and possessed a rough surface topography (Figure 1C). Biocompatibility testing confirmed the high cell viability on the PMMA fiber matrix (Figure 1D). Within 7 days of culture, a potentiation increase in the number of cells is observed similarly to the control substrates—glass and tissue culture polystyrene (TCPS). TCPS is the material on which cell cultures are classically conducted under laboratory conditions, while glass is a common substrate for cells in microscopic structural studies. Nevertheless, a notable increase in the cell population on PMMA fibers indicates the material’s excellent biocompatibility.^37^

The cells utilized the fibers as scaffolds for growth (Figure 1E), actively spreading and elongating between the fiber network. Osteoblasts and similar cell types displayed a propensity to utilize their surrounding microenvironment for 3D growth (Figure 1F). Even access to a single fiber proved sufficient for cells to enwrap and extend along its length successfully. Cells exhibited a preference for fiber substrates over glass, which offered easier accessibility. In cases where cells were initially growing on glass in a 2D culture, but fiber was present above, they took advantage of this scenario to initiate 3D growth (Figure 1G). Detailed observation of cell behavior and morphology in culture clearly revealed their robust inclination to expand spatially, leveraging the fiber scaffold for growth compared to a flat 2D culture.

Cells generated numerous adhesion sites to establish adhesion to the glass or fibers (Figure 1H). These sites corresponded to areas of significant protein accumulation in establishing strong cell attachment to the external environment. Detection of labeled specific proteins comprising these complexes enabled the localization of cell attachment sites by using fluorescence microscopy. The cells depicted in Figure 1H extended between two fibers. The distribution of paxillin (orange channel, Figure 1H) indicated the formation of multiple adhesion sites on both glass and fibers. Further magnification revealed distinct variations in the distribution of adhesion sites between glass (Figure 1I,J) and fiber (Figure 1K,L). During adhesion to glass, cells generated broad filopodia with adhesion sites at their tips (Figure 1H). In contrast, when binding to fiber surfaces, the adhesion sites were aligned linearly despite the availability of the entire fiber surface.

**Figure 2:**
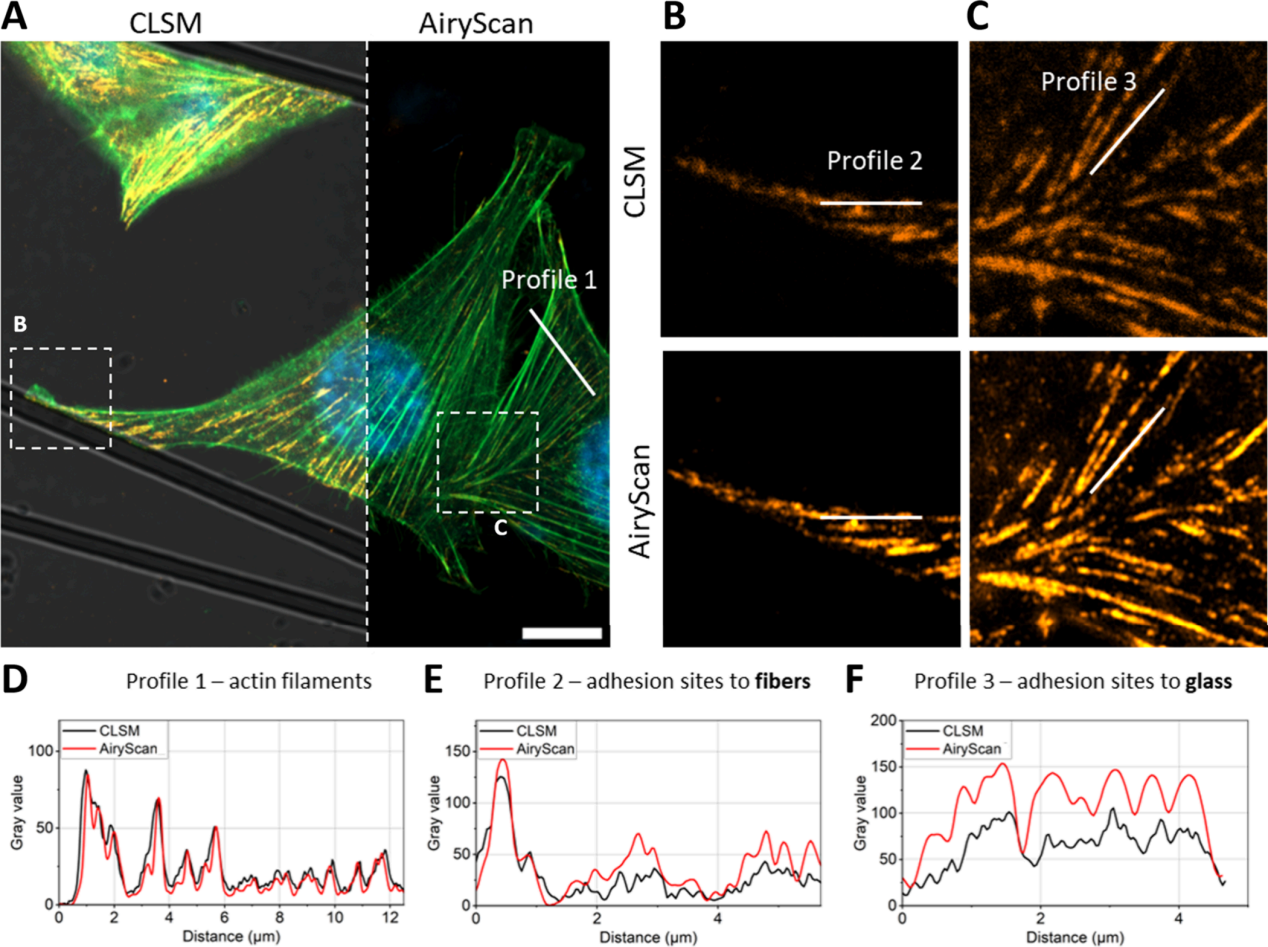
AiryScan imaging of osteoblast adhesion. (A) Comparison of CLSM and AiryScan images of cell stretched between two fibers. Scale bar: 10 μm. (B) Comparison of CLSM and AiryScan images of cell paxillin distribution in FA sites to fibers. (C) Comparison of CLSM and AiryScan images of cell paxillin distribution in focal FAs to glass. (D–F) Selected signal intensity profiles. Comparison between CLSM and AiryScan. Paxillin was stained with Alexa Fluor Plus 555 (orange), actin fibers were stained with Alexa Fluor 488 Phalloidin (green), and nuclei were counterstained with DAPI.

To quantitatively characterize the cell adhesion process to different materials, the special distribution of proteins was quantified using fluorescence labeling techniques and fluorescence microscopy employing the AiryScan method. Importantly, the AiryScan method enabled imaging of fluorescently labeled intracellular structures at higher resolution than traditional confocal microscopy (Figure 2A). AiryScan imaging provided a more precise differentiation of individual structures, which were not evident in confocal mode. The channel displaying the distribution of labeled paxillin (Figure 2B,C) exhibited that large adhesion sites identified in confocal mode comprised smaller local accumulations of paxillin distinguishable exclusively in AiryScan mode. Selected intensity profiles (Figure 2E,F) demonstrated enhanced resolution in the image due to increased signal-to-noise ratio. Profile 1 (Figure 2D) depicted the intensity profile of labeled actin within a cell fragment, serving as an example that, even for actin, increased resolution is observed in AiryScan mode.

**Figure 3:**
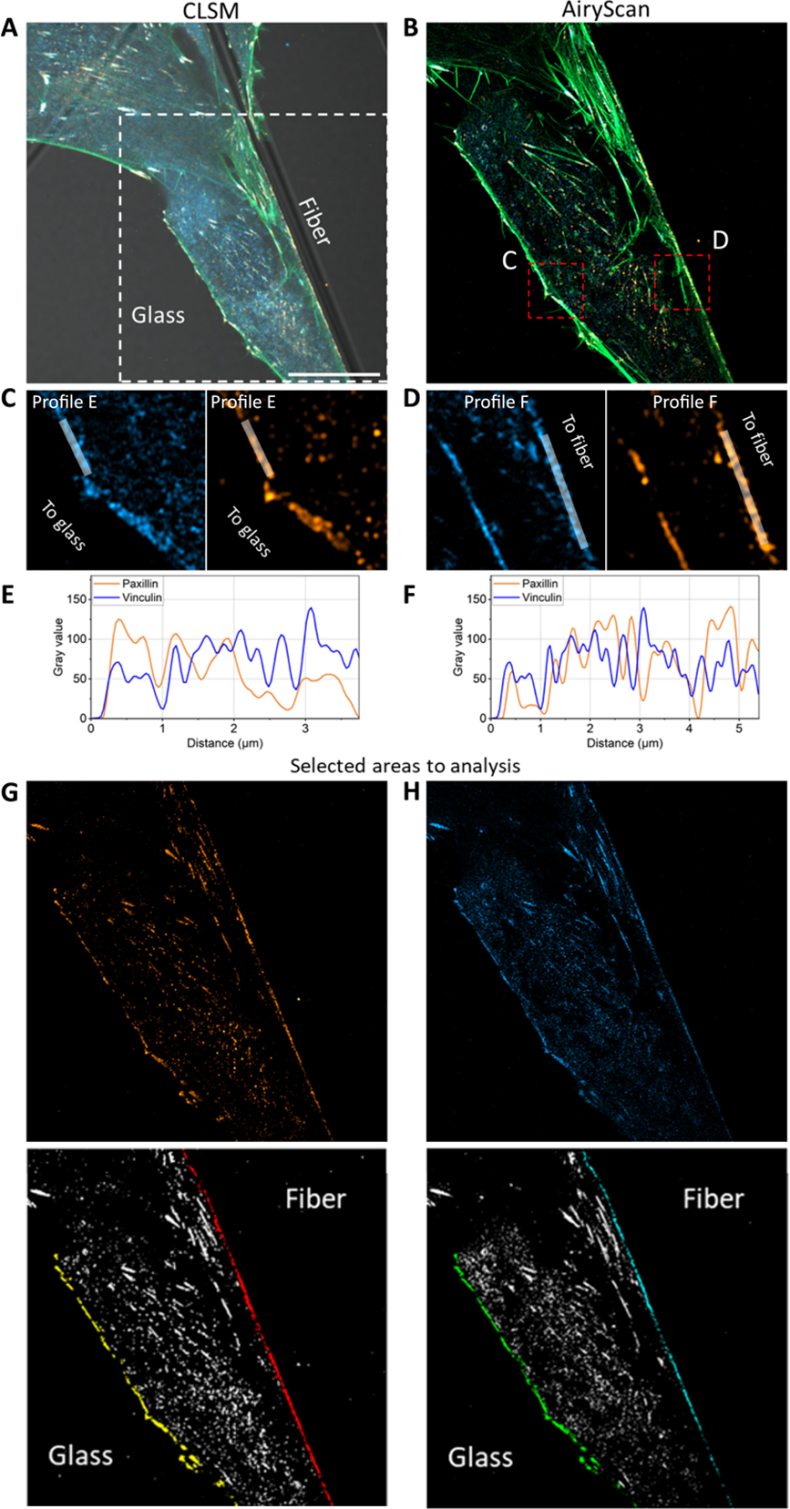
Selection of cell areas for adhesion analysis. (A) CLSM image of an osteoblast binding to fiber and glass. Scale bar: 20 μm with selected area imaged in AiryScan mode (B). (C) Vinculin (blue) and paxillin (orange) distribution in adhesion sites to glass. (D) Vinculin and paxillin distribution in adhesion sites to fibers. (E) Selected intensity profile from panel (C) in both channels for vinculin and paxillin. (F) Selected intensity profile from panel (D) in both channels for vinculin and paxillin. (G) Selection of areas for analysis in the image of paxillin distribution in the cell. Red: paxillin accumulation in adhesion sites to fibers; yellow: paxillin accumulation in adhesion sites to glass. (H) Selection of areas for analysis in the image of vinculin distribution in the cell. Blue: vinculin accumulation in adhesion sites to fiber; green: vinculin accumulation in adhesion sites to glass. Paxillin was stained with Alexa Fluor Plus 555 (orange), actin fibers were stained with Alexa Fluor 488 Phalloidin (green), and nuclei were counterstained with DAPI.

In this study, two essential proteins, vinculin and paxillin, known to contribute to developing adhesion sites, were simultaneously labeled. The cells were imaged using confocal microscopy to locate the fiber under transmitted light (Figure 3A) and the AiryScan to visualize signal distribution from accumulations of both proteins (Figure 3B). For the first time, it has been demonstrated that there are differences in the distribution of vinculin and paxillin accumulation at the adhesion sites. This observation was confirmed both at the sites of cell adhesion to glass and on the fibers (Figure 3C,D). The intensity profiles of the two labeled proteins at the adhesion site on glass (Figure 3E) differed significantly. Despite the crucial role of these proteins in adhesion site formation, their images did not overlap, and there was no strong correlation in the spatial localization of local intensity maxima. Similar observations were made in regions where the cell interacted with the fiber (Figure 3F). The spatial distribution of the two proteins lacked similarity, and there appeared to be a shift in space in one direction. In some instances, a local minimum of intensity in one channel (image of one protein distribution in the cell) correlated with maximum intensity in the other channel at both the glass and fiber binding sites. Importantly, this lack of spatial correlation suggests different recruitment mechanisms and dynamics of vinculin and paxillin to adhesion sites over time. Indeed, the advanced image analysis was employed to quantitatively describe the distribution of both proteins from super-resolution images, focusing on the morphologies of specific regions of interest. Two areas were defined for each channel corresponding to the recorded signal of the tagged protein: adhesion sites on glass (represented by yellow mask) and adhesion sites on the fiber (represented by red mask) for paxillin (Figure 3G) and a green mask for glass and blue mask for the fiber for vinculin (Figure 3H). By restricting the analysis to adhesion sites only, the influence of protein distribution inside the cell on the results was eliminated.

**Figure 4:**
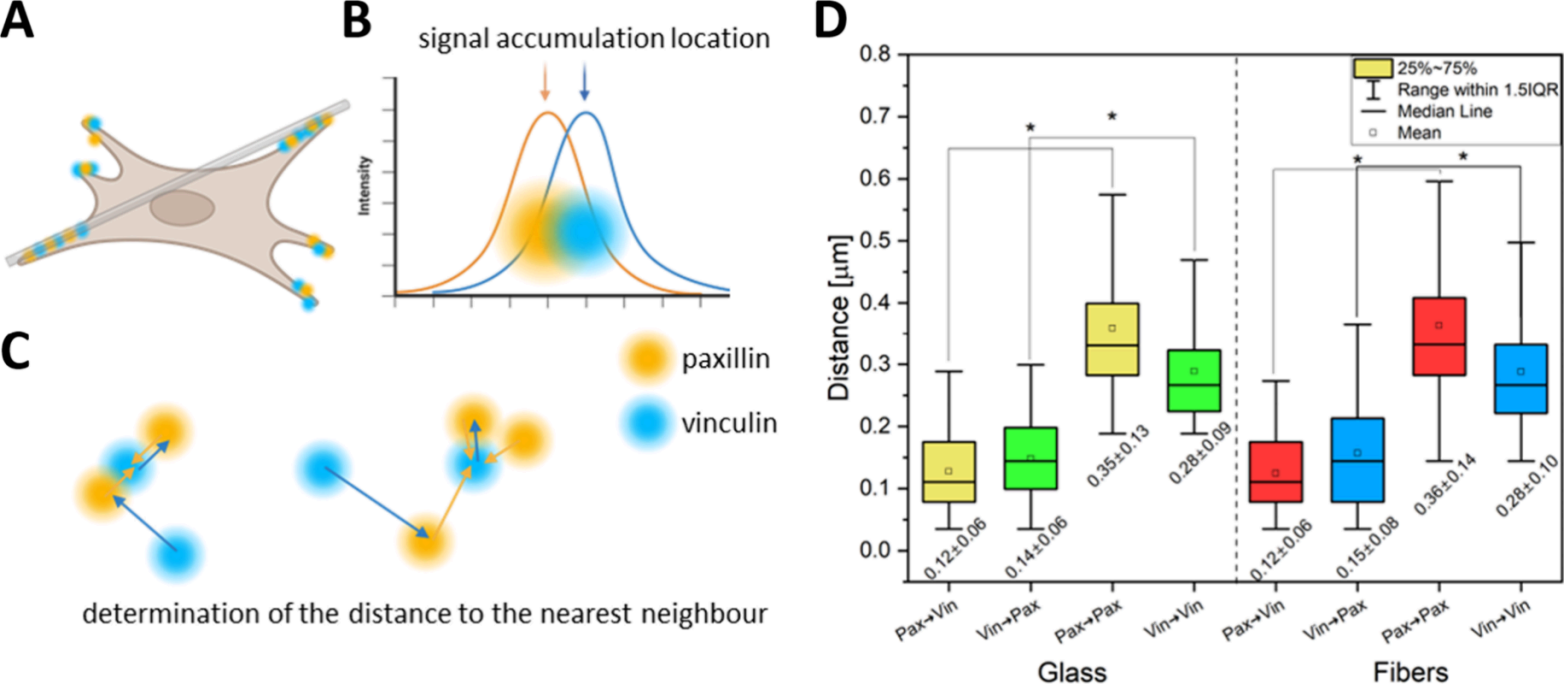
Analysis of the nearest neighbor. (A) Graphic of a cell stretched along a fiber with simultaneous bonding to glass. (B) Local maxima of signal intensities were located for both paxillin and vinculin channels. (C) Simplified rule for determining the distance to the nearest neighbor from the other channel. (D) Block diagrams of the distribution of distances to the nearest neighbor. Statistical significance was calculated with ANOVA, followed by Tukey’s post hoc test, *p < 0.05; error bars are based on standard errors.

First, within the defined areas of cell adhesion to glass and fibers (Figure 4A), local intensity maxima positions were determined for the labeled paxillin and vinculin signals (Figure 4B). For each paxillin local maximum, the distance to the nearest paxillin local maximum and the distance to the nearest vinculin accumulation representing a local maximum was calculated. A similar analysis was conducted for each recognized local maximum of the vinculin signal (Figure 4C). The distribution of these distances was illustrated in a diagram (Figure 4D). The results demonstrated that the distances from paxillin to vinculin were identical in both cases (0.12 ± 0.06 μm, respectively). When measuring the distance between vinculin and the closest paxillin, a slightly larger distance was observed (0.14 ± 0.06 and 0.15 ± 0.08 μm for glass and fiber, respectively), indicating that the local maxima of paxillin and vinculin were not precisely collocated but exhibited a slight shift approximately 100 nm concerning each other. In contrast, the distance analysis to the nearest neighbor of the same protein family (paxillin to paxillin and vinculin to vinculin) revealed that such foci were located further apart. In both cases, the paxillin foci exhibited a slightly broader spatial distribution than the local vinculin foci. The results suggest that vinculin and paxillin foci occur in close proximity to each other but do not precisely colocalize in space. The local distribution of paxillin and vinculin foci is similar when the cell binds to either glass or fiber.

### Cluster Size Analysis

In the context of microscopic image analysis, the DBSCAN algorithm (Density-Based Spatial Clustering of Applications with Noise) proves to be a valuable data clustering tool. DBSCAN excels at identifying clusters with varying densities, making it particularly well suited for the nuanced analysis of microscopic images. This algorithm adeptly handles situations where clusters exhibit different densities, enabling the precise characterization of spatial distribution patterns. Its inherent capability to tolerate noise effectively deals with isolated or scattered points, ensuring that they are excluded from the clustering analysis. This capability allows for a focused examination of meaningful clusters within the microscopic images. DBSCAN’s versatility extends to detecting clusters of arbitrary shapes, enabling the capture of intricate details within FA structures and facilitating exploration of their variations. Compared to k-means^38^ and similar methods, DBSCAN exhibits reduced sensitivity to parameter selection, making it more suitable for data-driven cluster identification.

**Figure 5:**
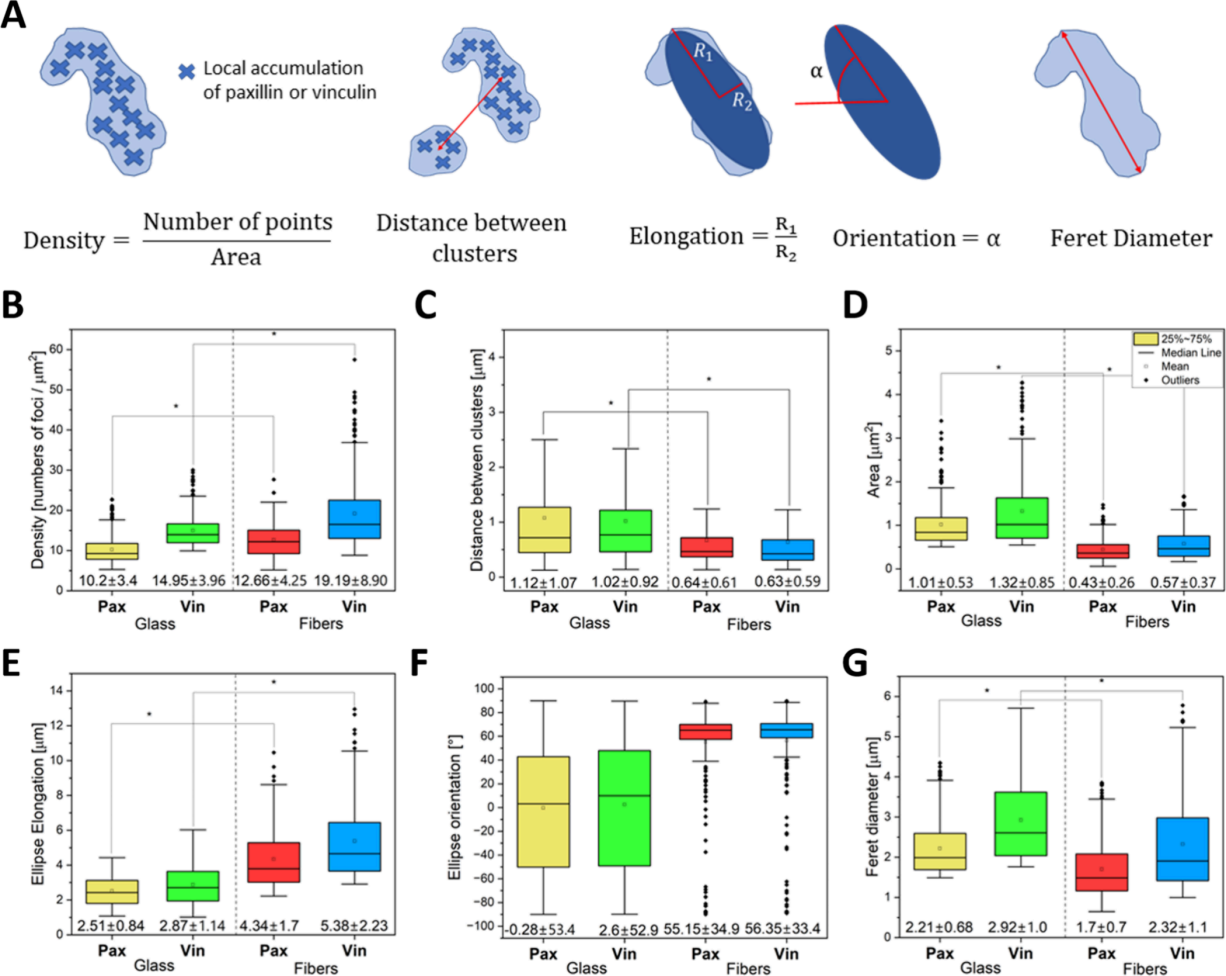
Results of the analysis of the identified clusters. (A) Explanation of the principle of subsequent analysis. (B–H) Block diagrams comparing the following distribution between paxillin and vinculin adhesion sites to fibers and glass. (B) Density points in clusters, (C) distance between clusters in the same channel, (D) area clusters, (E) ellipse elongation inscribed in the defined cluster, (F) ellipse orientation inscribed in the defined cluster, and (G) Feret diameter of the defined cluster. Statistical significance was calculated with ANOVA, followed by Tukey’s post hoc test, *p < 0.05; error bars are based on standard errors.

In the presented work, we examined the accumulation of vinculin and paxillin proteins at cell–substrate adhesion sites using microscopic images, as shown in Figure 3A. Our results indicate that both proteins play a crucial role in the formation of these adhesion sites, which are visible as local maxima of intensity in the images. We employed a super-resolution imaging technique to detect the local maxima in both channels and estimate the number of sites of accumulation for both proteins at binding sites. Additionally, based on the obtained microscopic images, the distribution of intensity maxima in both channels was determined using DBSCAN cluster analysis, which identified spots with a strong tendency to accumulate locally into larger clusters. The point density and area of these clusters were characterized using image morphology analysis (Figure 5A). Our findings show that when cells bind to polymer fibers, paxillin and vinculin form clusters containing 12.66 ± 4.25 and 19.19 ± 8.90 maxima per μm^2^, respectively (Figure 5B). On the other hand, when cells bind to glass, the average point densities of paxillin and vinculin are 10.20 ± 3.41 and 14.95 ± 3.96 maxima per μm^2^, respectively. The density of protein accumulation points in a cluster was determined as the quotient of the number of points in a cluster by its area. The obtained results show that in the adhesion clusters interacting with fibers, both proteins have more local clusters than in the adhesion to glass. Moreover, in both cases, the density of the local maxima of vinculin is higher than that for paxillin. Apart from the most common situation when one paxillin focus occurs together with a vinculin focus, we also observe a situation where two vinculin foci are next to one paxillin focus. An analysis of cluster distribution showed that at adhesion sites to glass, defined clusters lie at a greater distance from each other than analogous clusters at adhesion sites to fibers. The spatial distribution of clusters differs between cell adhesion to glass and adhesion to fibers. Specifically, clusters observed on glass substrates exhibit greater intercluster distances compared to clusters observed on fibers (Figure 5C).

We also found that the clusters of both vinculin and paxillin are more than twice as small when cells bind to fibers compared to glass (Figure 5D). The average sizes of the paxillin and vinculin clusters when cells bind to the polymer fiber are 0.43 ± 0.26 and 0.57 ± 0.37 μm^2^, respectively. In contrast, the average sizes of the analogous clusters involved in the binding process to glass are 1.01 ± 0.53 and 1.32 ± 0.85 μm^2^, respectively. To estimate the shape of the clusters, we described an ellipse on which the ratio of the two semiaxes was determined (Figure 5E), as well as its spatial orientation (Figure 5F). Our results show that the elongation of the ellipses for vinculin and paxillin is more significant when the cells bind to the polymer fibers, with values of 4.34 ± 1.70 for paxillin and 5.38 ± 2.23 for vinculin. When interacting with glass, the elongation of cluster-matched ellipses is two times lower for both paxillin and vinculin. Additionally, we analyzed the Feret diameter of the adhesion site clusters to gain insight into their size and shape (Figure 5G). The results support our previous findings that adhesion clusters to glass are larger and rounder than those to fibers. Despite the elliptical shape of the fiber’s adhesion clusters, they are still smaller in size than the glass adhesion clusters. Interestingly, when comparing the Feret diameter of vinculin and paxillin clusters in both cases of binding to glass and fiber, we observed that vinculin clusters have greater diameter. This observation is consistent with our previous analyses of cluster size and density (Figure 5B,C). Overall, these results highlight the importance of analyzing multiple parameters to understand the organization and behavior of adhesion site clusters comprehensively. Indeed, we provide insights into the accumulation of vinculin and paxillin at cell–substrate adhesion sites and their cluster formation. Our findings suggest that the size, density, and shape of these clusters are influenced by the substrate material, with differences observed between binding to fibers and glass underlining the importance not only of the gentry of the supports selected for tissue engineering. The surface area of the clusters for both proteins is larger in the case of cell adhesion to glass than fibers. In contact with glass, the cell produces adhesion clusters of even 2 to 6 μm^2^.

## Discussion

**Figure 6:**
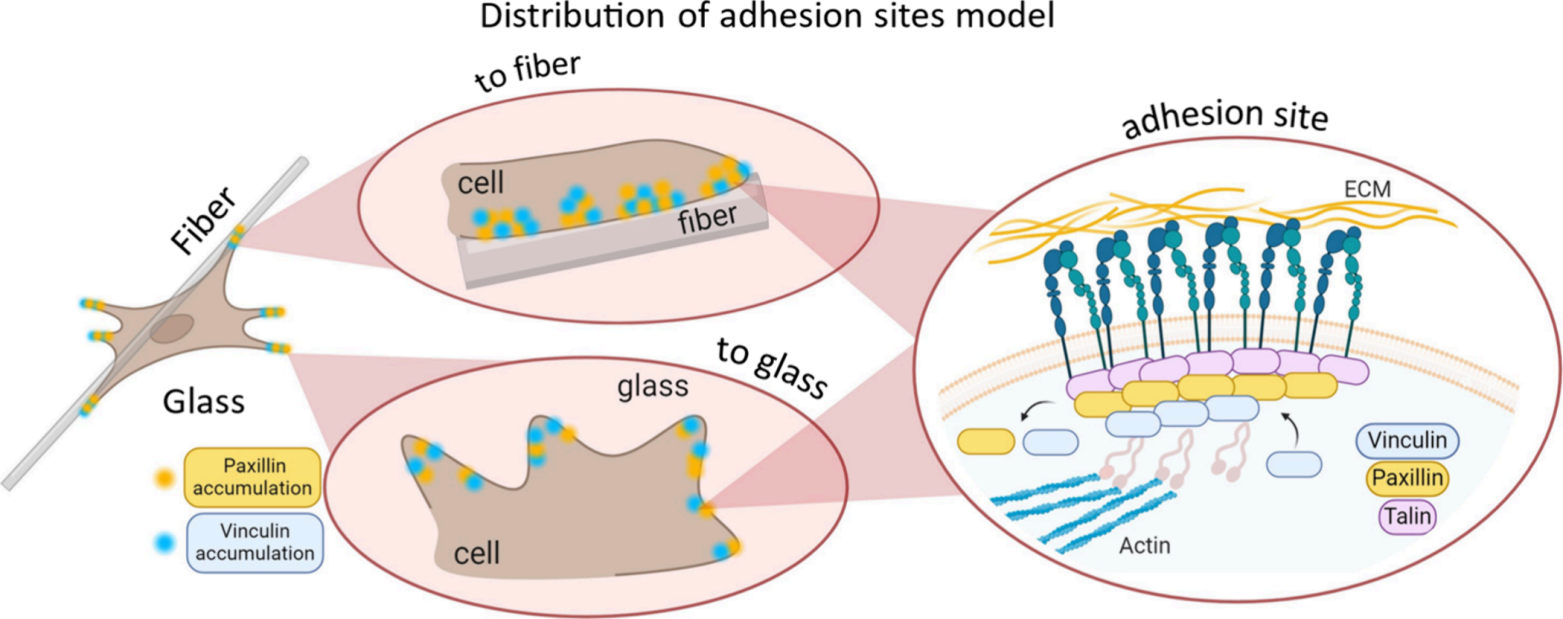
Model comparing the distribution of paxillin and vinculin involved in the formation of adhesion sites to glass and fibers.

Adhesion is one of the key properties of cells in research into material applications in regenerative medicine or tissue engineering. The imparting of appropriate mechanical and surface properties influences the dynamics and distribution of the resulting cell–material adhesion sites.^39^ Using high-resolution microscopy, it was possible to visualize the distribution of these adhesion sites.^28^ Advanced image analysis allowed quantitative characterization of the forming cell–substrate adhesion sites. Our work takes the pioneering step of visualizing the distribution of selected proteins engaged in cell adhesion to polymer fibers, widely used materials as a 3D scaffold for tissue engineering. The results are compared with the standard on glass experiments. We reveal significant differences not just in the distribution of adhesion complexes but also in the spatial displacements between local maxima of vinculin and paxillin. The distribution of paxillin and vinculin in cells can provide insights into the efficiency of the adhesion process to different extracellular materials. The organization and distribution of these proteins can reflect the strength and stability of cell adhesion. Compiling all the results, we propose visualizations in Figure 6 of the architecture of adhesion sites in cells for both types of external environments. This model indicates the differences between distribution of proteins involving the FA process on polymer fibers and the standard glass substrate, which is typically used in biological studies using the super-resolution microscopy techniques.^40,41^ Bertocchi et al.^41^ reveals that, upon activation, vinculin, guided by α-catenin, extends ∼30 nm, bridging cadherin–catenin and actin compartments and modulating actin regulator positions. This modular architecture enables vinculin to integrate mechanical and biochemical signals, selectively engaging cadherin–catenin complexes and regulating cell adhesions with the actomyosin system. Further research by Kanchanawong *et al.* extends the earlier contribution by proposing a layered model for the construction of adhesive complexes in cells based on high-resolution imaging, a model that is currently widely accepted.^25^ The influence of the geometry of the ECM on the behavior of cells and the efficiency of their adhesion to the substrate was addressed by Changede et al.^40^ The authors demonstrated with super-resolution microscopy that cell–matrix adhesions, mediated by integrins, actively sense the geometry and rigidity of extracellular environments, influencing important cellular processes. Our research is in line with the current trend focusing on the dynamics and architecture of complexes involved in cell adhesion. Our findings are adding the next level of understanding to the previously reported results in this field, as we show for the first time the behavior of specific proteins during the cell adhesion process to polymer fibers, commonly utilized in tissue engineering as cell scaffolds. Our study aims to elucidate the architecture of the adhesion process when cells interact with polymer fiber scaffolds. Currently, we are able to control surface properties, charges and geometry, all of which greatly impact how cells adhere and, consequently, influence the tissue regeneration process.^42−45^

We compared the process of osteoblast attachment to glass, a classical system used in cell imaging, and polymer fibers widely used in tissue engineering. In both cases, there is a shift in the local maxima of the paxillin and vinculin signals with respect to each other. This agrees with previous knowledge that both proteins are crucial in the coformation of adhesion sites. The shift can be due to the dynamics of creating such sites. Both proteins are located in different layers by which the local site of maximum accumulation can be shifted. Using cluster analysis with the DBSCAN algorithm, both proteins’ quantized signal was clustered. The results show that the clusters of adhesion sites to glass are larger and rounder and contain a lower density of accumulated protein (Figure 5). In the case of adhesion to fibers, the clusters formed are more elliptical and smaller and have higher protein density (Figure 5). The size and density of FAs, which are influenced by paxillin and vinculin, can reflect the strength and stability of cell adhesion. Efficient adhesion is often associated with well-organized and larger FAs characterized by increased paxillin and vinculin accumulation. In contrast, a fragmented or sparse distribution of FAs can suggest weaker or less efficient adhesion. Mature FAs are characterized by a high concentration of paxillin and vinculin, indicating a stronger attachment between cells and the extracellular material. Immature or less efficient adhesions can exhibit a more diffuse distribution of paxillin and vinculin. The dynamics of FAs, including their assembly, disassembly, and turnover rates, can affect adhesion efficiency. Efficient adhesion is often associated with a balanced turnover of FAs, with continuous remodeling and replacement of adhesion sites. The distribution of paxillin and vinculin can provide insights into the turnover rate and stability of FAs. Analysis of the angular orientation of the clusters confirms that the clusters of adhesion to glass are oriented in different directions. In contrast, the orientation of the adhesion clusters to fibers is very consistent. The clusters line up one behind the other.

The 3D architecture of fibrous scaffolds closely mimics that of the native ECM, providing a more physiologically relevant environment for cell growth and adhesion. Thus, any analysis of cell adhesion on polymer fibers is more relevant in translating the results for further in vitro experiments and in vivo tests than from glass. The samples with fibers introduce complexities for sample preparation and cell staining; however, the results are related to the unique topology and surface chemistry, influencing the distribution and accumulation of adhesive proteins. The fiber structure of polymer scaffolds can indeed control and influence cell adhesion dynamics, including osteoblast adhesion, to the scaffold. The architecture, arrangement, and properties of the fibers within the scaffold can play a significant role in regulating cell–scaffold interactions. Designing and manipulating the fiber structure of polymer scaffolds allow to control and optimize cell adhesion dynamics, including osteoblast adhesion. This can lead to improved cellular interactions and tissue integration and ultimately enhance the functionality and performance of the scaffold in tissue engineering and regenerative medicine applications.

## Conclusions

We show a novel approach to studying the distribution of specific proteins involved in cell adhesion to polymer fibers as a model used in tissue engineering scaffolds. Noteworthily, we indicate the variations in the distribution of adhesion complexes and highlight significant differences in the spatial displacements between local maxima of vinculin and paxillin on polymer fibers and the typically studied glass. Our study quantitatively compares osteoblast adhesion processes on glass and polymer scaffolds, marking the first detailed analysis on a 3D model incorporating polymer fibers into the tissue scaffold. The results are primarily compared to extensively studied glass, with established procedures for cell staining and observations on glass. However, complexity is added when dealing with polymer fibers. Directly studying cell–material interactions is crucial for translating fundamental biomaterial research in the medical field. Novel methods and protocols have been developed for this research employing vinculin and paxillin proteins. The tool integrates super-resolution imaging and spatial analysis for further refinement, describing osteoblast adhesion to polymer scaffolds with varying surface properties. In the long term, it is planned to use the developed method to analyze differences in the adhesion of osteoblasts to other materials, including polymer fibers with diverse mechanical or surface properties. The opportunity to characterize adhesion at the molecular level will be an essential step toward understanding the adhesion process and how it changes depending on the type of substrate with which the cells interact.

## Materials and Methods

### Preparation of Electrospun Fibers

To obtain a 12 wt % solution, poly(methyl methacrylate) (PMMA, *M*_w_ = 350 000 g·mol^–1^, Sigma-Aldrich, UK) was dissolved in *N*,*N*-dimethylformamide (DMF, Sigma-Aldrich, UK) (purity, ≥99.8%). The solution was stirred at 700 rpm for 2 h on a hot plate set at 55 °C (IKA RCT Basic, Germany). PMMA fibers were produced via an electrospinning machine (apparatus EC-DIG with climate control, IME Technologies, The Netherlands) at *T* = 25 °C and 40% relative humidity. A voltage of 12 kV was applied to the needle kept at a distance of 15 cm from the grounded drum rotating at 2000 rpm.^46^ The inner diameter of the needle was 0.51 mm, and the outer diameter was 0.82 mm. The flow rate was set to 4 mL·min^–1^. The electrospinning time was 30 s for all samples. The samples were directly deposited on a 10 × 10 glass wafer/substrate (cover glass, Epredia, USA) for CLSM analyses.^47^ The average fiber diameter values were calculated from 100 measurements based on SEM images, and the error was based on the standard deviation. The fiber diameter was analyzed using ImageJ software (version 1.51, Fiji, USA).

### Cell Culture and Proliferation Assay

Human osteosarcoma-derived osteoblast-like cell line MG63 (ECACC, Sigma-Aldrich, Dorset, UK) was used in the studies. A coverslip containing dozens of PMMA fibers was placed in 24-well plates and sterilized for 30 min in UV light. Cells were seeded on the sample at the concentration of 2 × 10^4^ cells per mL for 3 days. The cellular medium consisted of Dulbecco’s modified Eagle medium (Thermo Fisher Scientific, US) supplemented with 10% addition of fetal bovine serum (FBS), 2% antibiotics (penicillin/streptomycin), 1% amino acids, and 1% l-glutamine (Sigma-Aldrich, UK). The culture was grown under standard conditions, i.e., at *T* = 37 °C, RH = 95%, and 5% CO_2_. Cell proliferation was assessed using CellTiter Blue (Promega, USA) at 1, 3, and 5 days of HaCaT cell cultivation on PMMA fibers, glass, and TCPS. Two replicates per sample type were conducted at each time point. Following the specified incubation period, the culture medium was removed, and 80 μL of CellTiter Blue reagent, along with 400 μL of fresh cell culture medium, was added. The mixture was then incubated for 4 h at 37 °C in a 5% CO_2_-humidified atmosphere. Subsequently, 100 μL of the reaction solution was transferred to a new 96-well plate in quadruplicate, and fluorescence was measured (excitation/emission, 560/590 nm).

### Staining Procedure

Cells were fixed in 4% paraformaldehyde solution for 15 min at room temperature (23 °C). Fixed cells were permeabilized with 0.1% Triton X-100 (Sigma-Aldrich, USA) in PBS for 10 min at 22 °C, followed by three washes with PBS and blocking in 3% bovine serum albumin solution (BSA, Sigma-Aldrich, UK) for 1 h. To visualize actin filaments, cells were incubated for 1 h at 23 °C with Alexa Fluor 633 Phalloidin (Thermo Fisher, USA).

Cells were incubated with the mixture of primary antibodies: rabbit anti-Paxillin (1:100, ab32084, Abcam) and mouse anti-Vinculin (1:100, ab130007, Abcam) diluted in PBS with 0.1% BSA for 1 h. Afterward, the cells were washed 3 times in PBS for 15 min, followed by incubation with the mixture of secondary antibodies: Alexa Fluor Plus 405-conjugated antimouse IgG (1:1000, A48255, Thermo Fisher, USA) and Alexa Fluor 555-conjugated antirabbit IgG (1:1000, A21428, Thermo Fisher, USA). Nuclear DNA was stained with 4′,6-diamidino-2-phenylindole (DAPI, Sigma-Aldrich, UK) for 5 min. After staining, the sample was washed three times (15 min each) with PBS. Slides were mounted with Vectashield antifade mounting media (Merck, USA).

### Confocal Microscopy and AiryScan

Confocal and in AiryScan mode imaging was performed by using a Zeiss LSM 900 confocal microscope (Carl Zeiss Microscopy GmbH). Images were acquired using ZEN 3.1 software (Carl Zeiss Microscopy GmbH) and processed using ImageJ 1.53v (National Institutes of Health, Bethesda, Maryland, USA). The following microscopy parameters were used for both confocal and AiryScan modes: Plan-Apochromat 63×/1.4 Oil DIC M27; excitation, 405, 561, and 633 nm (diode lasers); emission detection bands, 400–600 nm for Alexa Fluor, 405 and 450–545 nm for Alexa Fluor 488 coupled with Phalloidin, and 540–700 nm for Alexa 555. Registration was performed in sequential mode, 16-bit dynamic range. An AiryScan2 detector was used for AiryScan imaging.

### Image Analysis

#### Defining Areas

The distribution of vinculin and paxillin in the areas of cell adhesion to PMMA fibers or glass was imaged in separate channels. To precisely determine the sites of cell adhesion to the fibers, PMMA fibers were recorded under the transmitted light channel. Data analysis was conducted semiautomatically using macros developed in ImageJ software. The fluorescence images were normalized using the Statistical Dominance Algorithm (SDA).^48^ SDA calculates the number of pixels based on their relation to the central point of the neighborhood, enabling the classification of points (peak, valley, and slope) and reducing the impact of noise or uneven illumination in the image results. It generates an output image that represents the statistical dominance of points over their neighborhoods, facilitating effective point classification (Figure S1). In the paxillin distribution image, the areas where the cell interacts with PMMA fibers and glass were manually defined. Similarly, areas were defined for the image of vinculin distribution in the cell (Figure 3G). Glass adhesion sites were selected at the periphery of the cell where the cell was stretched, while the adhesion areas of the cell to the PMMA fiber were selected based on the transmitted light channel where both the cell and fiber were visible. Further analysis was conducted only on the defined areas in both channels.

#### Finding Maxima and Determining Regions

The defined areas of adhesion to the fiber or to the glass were normalized. Local maxima were determined using the available tools in ImageJ. The MorphoLibJ^49^ collection of mathematical morphology methods and plugins for ImageJ were used to determine the areas for each maximum and their subsequent clustering according to DBSCAN analysis.

#### Nearest-Neighbor Analysis (NN)

Local peaks of signal intensity were identified using macros prepared in ImageJ. Each peak corresponds to a local accumulation of either vinculin or paxillin at an adhesion site. Nearest-neighbor (NN) analysis was performed by estimating the minimum distance from each point to all other points, which could belong to the same group of molecules (autoanalysis) or to a different group. The distribution of distances to the nearest neighbor was presented using a box plot.

#### DBSCAN Analysis

Based on the obtained map of points representing local maxima of signal intensity from paxillin and vinculin, Density-Based Spatial Clustering of Applications with Noise (DBSCAN) analysis was performed. The DBSCAN algorithm is a well-known density-based clustering approach that utilizes the proximity of data points to form clusters. By considering the density of the data or how closely the points are situated, this algorithm excludes points that are outside the dense regions and treats them as noise or outliers. As a result, DBSCAN is an ideal candidate for detecting outliers and clustering data with diverse shapes and sizes. The algorithm employs a parametric method that relies on two key parameters: epsilon (eps) and minimum points (min_pts). Epsilon (eps) represents the radius of the neighborhood around a data point, while min_pts represents the minimum number of data points required in the vicinity of a specific point to establish a cluster (Figure S2). The parameters were selected based on a learning set of 10 images. Then, based on the selected parameter values, the analysis was performed on the remaining data.

Figures 1, 4, and 6 were created with BioRender.com

## Supporting Information Available

Figure S1: effect of SDA on microscopic AiryScan images; Figure S2: principle of the algorithm (PDF)
