# Supplementary material for: Cell–Material Interplay in Focal Adhesion Points
Source: ACS Appl Mater Interfaces. 2024 Feb 14;16(8):9944–55. doi: 10.1021/acsami.3c19035 (PMC10910443; doi:10.1021/acsami.3c19035)
Supplement: Supplementary file 1 — am3c19035_si_001.pdf [file am3c19035_si_001.pdf]

Supporting Information for

Cell-Material Interplay in Focal Adhesion Points

Krzysztof Berniak[1], *Daniel P. Ura*[1], *Adam Piórkowski*[2], *Urszula Stachewicz*[1]*

[1]Faculty of Metals Engineering and Industrial Computer Science, AGH University of Krakow, al. A. Mickiewicza 30, Krakow 30-059, Poland

[2]Department of Biocybernetics and Biomedical Engineering, AGH University of Krakow, al. A. Mickiewicza 30, Krakow 30-059, Poland.

*E-mail: ustachew@agh.edu.pl

This file includes:

Figure S1. Effect of SDA algorithm on microscopic AiryScan images.

Figure S2. Principle of the algorithm.

Figure S1.

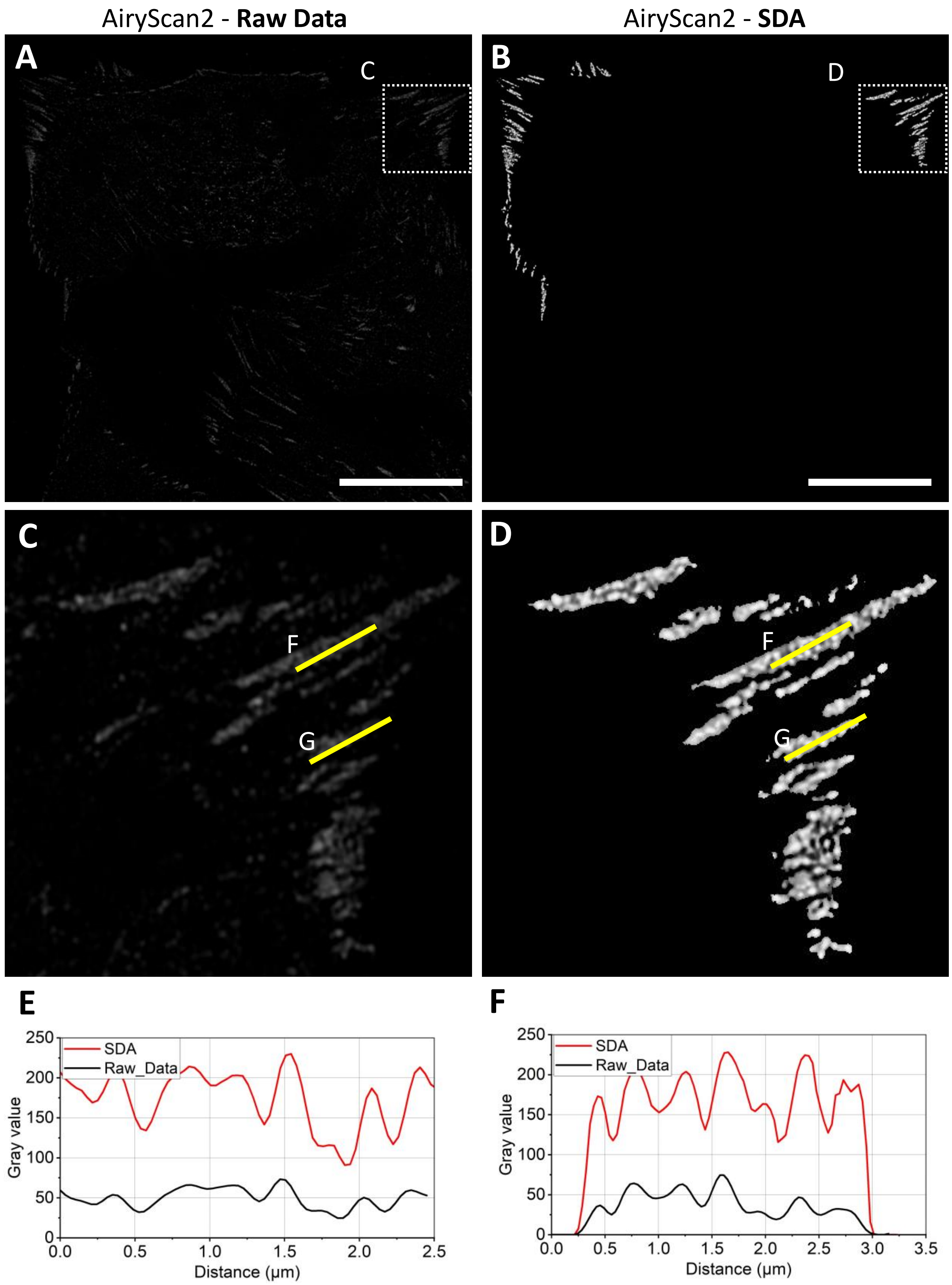

Figure S1. Effect of SDA algorithm on microscopic AiryScan images. (**A**) AiryScan image of paxillin distribution in cells. (**B**) AiryScan image of paxillin after running the SDA algorithm and selecting areas of cell adhesion to the environment**.** Scale bar: 20 µm. (**C, D**) enlargements

of the selected area recorded on AiryScan images before and after the SDA algorithm operation, respectively. (**E, F**) Comparison of selected intensity profiles on AiryScan images before and after the SDA algorithm operation, respectively.

Figure S2.

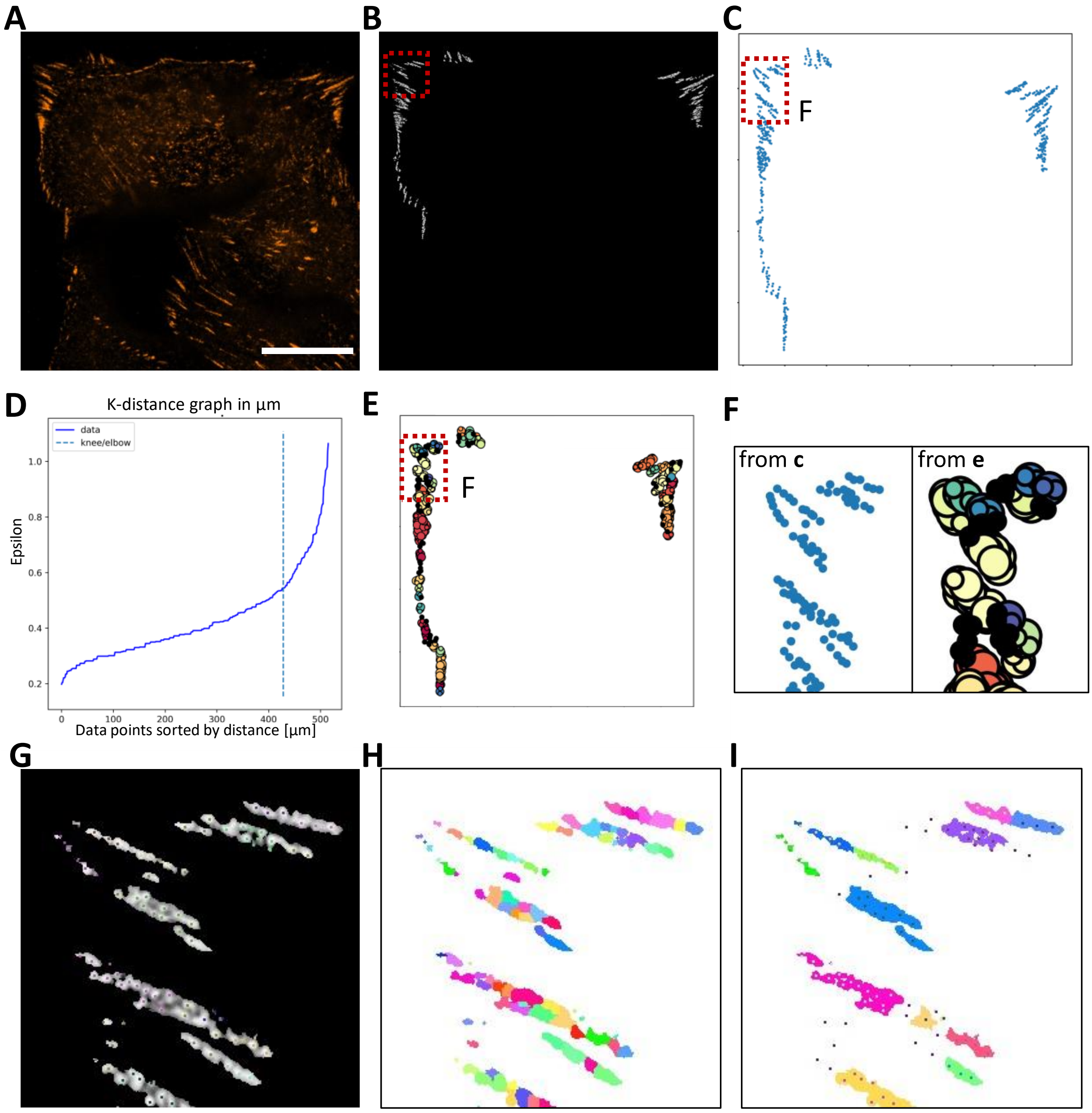

Figure S2. Principle of the algorithm (**A**) CLSM image of paxillin distribution in cells. (**B**) AiryScan image of paxillin after running the SDA algorithm and selecting areas of cell adhesion to the environment**.** Scale bar: 20 µm. (**C**) map of the location of recognized local intensity maxima. (**D**) Determination of the value of the epsilon parameter in the DBSCAN algorithm based on the inflection of the K-distance graph. (**E**) The result of the DBSCAN algorithm. Large dots are cores, smaller dots are reachable for cores. Black dots are outliners - they do not belong to clusters. (**F**) Zoom in on a selected section from the c and e diagrams. (**G**) A fragment of image b with the points applied. Points of the same color belong to one cluster. **H** For each recognized point, the area belonging to it was determined based on the image b. (**I**) Areas belonging to points from the same cluster have been merged into one object. black dots are outliners that do not belong to clusters. The size of the images (**G-I**) is 9µm.
